# Supplementary material for: Applying the S-O-R model to explore impulsive buying behavior driven by influencers on social commerce websites
Source: PeerJ Comput Sci. 2025 Sep 11;11:e3113. doi: 10.7717/peerj-cs.3113 (PMC12453796; doi:10.7717/peerj-cs.3113)
Supplement: Supplemental Information 1 [file peerj-cs-11-3113-s001.docx]

**Appendix A: Research Questionnaire Items - Construct Measurement Items and Reference Sources**

**Price Discount (DP) Reference:** Chen & Yao (2018)

- I buy things because merchants sell them at discounted prices, not because I need the item
- Because of discounts, I buy more things
- I like to find discount posts from influencers on social commerce websites

**Scarcity (SCA) Reference:** Chen & Yao (2018)

- I worry about limited product quantities
- When I see "sold out," I feel anxious
- I think a limited edition product will attract many people to buy
- I think the current supply of limited products is minimal

**Review Quality (RQ) Reference:**Xu et al. (2020)

- I think the reviews on social commerce websites are complete
- I think the reviews on social commerce websites are accurate
- I think the reviews on social commerce websites are trustworthy
- I think the reviews on social commerce websites are objective

**Observational Learning (OL) Reference:** Xu et al. (2020)

- I can easily observe that many people purchase products introduced by influencers on social commerce websites
- I observe that posts about products introduced by influencers on social commerce websites have high sales volumes
- I observe that many people ask where to buy items displayed by influencers
- I observe that products introduced by influencers are quickly sold out

**Positive Affect (PA) Reference:** Xu et al. (2020)

- When shopping on social commerce websites, I feel excited
- When shopping on social commerce websites, I feel emotionally aroused
- When shopping on social commerce websites, I feel satisfied
- When shopping on social commerce websites, I feel happy

**Social Presence (SP) Reference:** Wang et al. (2021)

- When watching influencer live streams, I have a social feeling
- When watching influencer live streams, I can feel contact with people
- When watching influencer live streams, I can feel the enthusiasm of others
- When watching influencer live streams, I can feel the presence of other viewers
- When watching influencer live streams, I can exchange information with other viewers

**Flow State (FS) Reference:** Ming et al. (2021); Wang et al. (2021)

- When watching live streams, I don't want to take my eyes off the stream
- When watching live streams, I ignore what's happening around me
- When watching live streams, I find it very interesting
- When watching live streams, I feel time passes quickly

**Sense of Belonging (SOB) Reference:** Teo et al. (2003); Lin (2008); Zolkepli & Kamarulzaman (2015)

- I have a strong sense of belonging to social commerce websites
- I like being a member of social commerce websites
- I completely trust other people on social commerce websites

**Entertainment (EN) Reference:** Ki et al. (2020)

- I think the influencers I follow are interesting
- I think the influencers I follow are funny
- I think the influencers I follow are humorous
- When I'm sad, I think the influencers I follow can make me feel happy

**Informativeness (IM) Reference:** Ki et al. (2020)

- I use the content posted by the influencers I follow as an information source
- I find the content posted by the influencers I follow very practical
- I learn from the content posted by the influencers I follow

**Emotional Attachment (EA) Reference:** Aw & Labrecque (2020); Ki et al. (2020)

- If there are stories about the influencers I follow in newspapers or magazines, I will read them
- If the influencers I follow disappeared from my life forever, I would be very sad
- I look forward to watching posts from the influencers I follow on social commerce websites
- When the influencers I follow show me their views on luxury brands, it helps me make my own decisions about the brand
- I have an emotional attachment to the influencers I follow

**Impulse Buying Behavior (IBB) Reference:** Chen & Yao (2018); Xu et al. (2020); Ming et al. (2021)

- When using social commerce websites, I buy things I didn't intend to buy
- When using social commerce websites, I spend more money on products than I expected
- When using social commerce websites, if I see discount prices, I make impulsive purchases
- When using social commerce websites, I am somewhat reckless when buying products
- When using social commerce websites, my current emotions affect my purchase intention
- When using social commerce websites, I often buy products displayed by the influencers I follow, even when I don't need them

**References**

Aw, E. C.-X., & Labrecque, L. I. (2020). Celebrity endorsement in social media contexts: understanding the role of parasocial interactions and the need to belong. *Journal of Consumer Marketing*, *37*(7), 895-908.

Chen, C.-C., & Yao, J.-Y. (2018). What drives impulse buying behaviors in a mobile auction? The perspective of the Stimulus-Organism-Response model. *Telematics and Informatics*, *35*(5), 1249-1262.

Ki, C.-W. C., Cuevas, L. M., Chong, S. M., & Lim, H. (2020). Influencer marketing: Social media influencers as human brands attaching to followers and yielding positive marketing results by fulfilling needs. *Journal of Retailing and Consumer Services*, *55*, 102133.

Lin, H.-F. (2008). Determinants of successful virtual communities: Contributions from system characteristics and social factors. *Information & Management*, *45*(8), 522-527.

Ming, J., Jianqiu, Z., Bilal, M., Akram, U., & Fan, M. (2021). How social presence influences impulse buying behavior in live streaming commerce? The role of SOR theory. *International Journal of Web Information Systems*, *17*(4), 300-320.

Teo, H.-H., Chan, H.-C., Wei, K.-K., & Zhang, Z. (2003). Evaluating information accessibility and community adaptivity features for sustaining virtual learning communities. *International Journal of Human-Computer Studies*, *59*(5), 671-697.

Wang, H., Ding, J., Akram, U., Yue, X., & Chen, Y. (2021). An empirical study on the impact of e-commerce live features on consumers’ purchase intention: From the perspective of flow experience and social presence. *Information*, *12*(8), 324.

Xu, X. (2020). Examining consumer emotion and behavior in online reviews of hotels when expecting managerial response. *International Journal of Hospitality Management*, *89*, 102559.

Zolkepli, I. A., & Kamarulzaman, Y. (2015). Social media adoption: The role of media needs and innovation characteristics. *Computers in human behavior*, *43*, 189-209.
