# Supplementary material for: Applying the S-O-R model to explore impulsive buying behavior driven by influencers on social commerce websites
Source: PeerJ Comput Sci. 2025 Sep 11;11:e3113. doi: 10.7717/peerj-cs.3113 (PMC12453796; doi:10.7717/peerj-cs.3113)
Supplement: Supplemental Information 3 [file peerj-cs-11-3113-s003.pdf]

表 1、研究問卷之問項

構念	衡量問項	參考來源
價格折扣（DP）	我買東西是因為商家以折扣價出售，而不是因為我需要這項物品	Chen&Yao（2018）
	因為打折，我會買更多的東西	
	我喜歡在社交商務網站裡找網紅發文的折扣	
稀缺性（SCA）	我擔心商品數量有限	Chen&Yao（2018）
	當我看到"售完"，會感到焦慮	
	我覺得一個限量版的商品會引起很多人的購買	
	我覺得目前限量商品的供應量很小	
評論品質（RQ）	我覺得社交商務網上的評論是完整的	Xu et al.（2020）
	我覺得社交商務網站上的評論是正確的	
	我覺得社交商務網站上的評論是可信的	
	我覺得社交商務網站上的評論是客觀的	
觀察學習（OL）	我容易觀察到社交商務網站上網紅介紹的商品被很多人購買	Xu et al.（2020）
	我觀察到社交商務網站上網紅介紹的商品貼文銷售量都很高	
	我觀察到許多人會詢問網紅展示的物品該去哪裡買	
	我觀察到網紅介紹的商品很快就被買完	
正面情緒（PA）	在社交商務網站購物時，我會感到興奮	Xu et al.（2020）
	在社交商務網站購物時，我會感到情緒激昂	
	在社交商務網站購物時，我會感到滿足	

構念	衡量問項	參考來源
	在社交商務網站購物時，我會感到快樂	
社交臨場感（SP）	在觀看網紅直播時，我會有一種社交的感覺	Wang et al.（2021）
	在觀看網紅直播時，我可以感受到與人接觸的情況	
	在觀看網紅直播時，我可以感受到其他人的熱情	
	在觀看網紅直播時，我可以感受到其他觀看者的存在	
	在觀看網紅直播時，我可以與其他觀看者交換資訊	
心流（FS）	看直播時，眼睛都不想離開直播	Ming et al.（2021）；Wang et al.（2021）
	看直播時，忽略了身邊發生的事	
	看直播時，覺得很有趣	
	看直播的時候，感覺時間過得很快	
歸屬感（SOB）	我對社交商務網站有強烈的歸屬感	Teo et al.（2003）；Lin（2008）；Zolkepli & Kamarulzaman（2015）
	我喜歡成為社交商務網站的一員	
	我完全信任社交商務網站中的其他人	
娛樂性（EN）	我覺得我所關注的網紅很有趣	Ki et al.（2020）
	我覺得我所關注的網紅很搞笑	
	我覺得我所關注的網紅很幽默	
	當我傷心時，我覺得我所關注的網紅能使我感到開心	
資訊性	我使用我所關注的網紅的發文內容作為資訊來	

構念	衡量問項	參考來源
（IM）	源	Ki et al.（2020）
	我發現我所關注的網紅的發文內容很有實用性	
	我會學習我所關注的網紅的發文內容	
情感依戀（EA）	如果報紙或雜誌上有關於 我所關注的網紅的故事，我會閱讀	Aw & Labrecque（2020）；Ki et al.（2020）
	如果我關注的網紅永遠從我的生活中消失，我會很難過	
	我期待在社交商務網站上觀看我所關注的網紅的發文	
	當我所關注的網紅向我展示她對這個奢侈品牌的看法時，這有助於我對這個品牌做出自己的決定	
	我對我關注的網紅有感情上的依戀	
衝動購買行為（IBB）	使用社交商務網站時，我購買了我不打算購買的東西	Chen&Yao（2018）；Xu et al.（2020）；Ming et al. （2021）
	使用社交商務網站時，我購買商品的金額比我預期的多	
	使用社交商務網站時，如果我看到折扣價，我會一時衝動購買	
	使用社交商務網站時，買商品時我有點魯莽	
	使用社交商務網站時，我現在的情緒會影響我的購買意願	
	使用社交商務網站時，我常會購買我關注的網紅所展示的商品，即使我不需要	

表 2、研究變數與操作型定義

構念	變數定義	題項數	參考文獻
價格折扣（DP）	網紅貼文中提供降低商品的零售成本以吸引消費者。	3	Chen&Yao（2018）
稀缺性（SCA）	通過限製商品的數量或時間向消費者傳達購買該商品的可能性很低的程度。	4	
評論品質（RQ）	在網紅貼文中消費者對評論的知覺訊息品質的程度	4	Xu et al.（2020）
觀察學習（OL）	消費者觀察其他消費者在網紅貼文中按讚、分享和評論的數量及在網站上購買商品狀況的程度	4	Xu et al.（2020）；Zafar et al. 2021
社交臨場感（SP）	在網紅直播時的感受如同面對面交流一樣的程度。	5	Wang et al.（2021）
歸屬感（SOB）	在網紅直播時，對社群的認同，認為自己是群體的一員	3	Hsu（2020）
娛樂性（EN）	網紅本身使消費者感到娛樂或有趣的程度。	4	Chen & Lin（2018）
資訊性（IM）	消費者從網紅那得到有用資訊的程度。	3	
心流（FS）	消費者全神貫注於直播的心理狀態的程度。	4	Chen & Lin（2018）；Wang et al.（2021）
正面情緒（PA）	消費者在瀏覽網紅貼文時受到環境刺激感到開心的程度。	4	Chan et al.（2017）
情感依戀	消費者對網紅感知強烈情感的程	5	Aw & Labrecque

構念	變數定義	題項數	參考文獻
（EA）	度。		（2020）；Chen et al.（2021）
衝動購買行為（IBB）	消費者在購物場景中接受外部刺激後，表現出強烈的情緒反應，購買原本不打算購買的商品的行為。	6	Chen&Yao（2018）；Xu et al.（2020）；Ming et al. （2021）
